# Antimicrobial Resistance and Virulence Potential of Bacterial Species from Captive Birds of Prey—Consequences of Falconry for Public Health

**DOI:** 10.3390/ani14060856

**Published:** 2024-03-11

**Authors:** Rita Magalhães, Luís Tavares, Manuela Oliveira

**Affiliations:** 1 CIISA—Centre for Interdisciplinary Research in Animal Health, Faculty of Veterinary Medicine, University of Lisbon, 1300-477 Lisbon, Portugalltavares@fmv.ulisboa.pt (L.T.); 2 AL4AnimalS—Associate Laboratory for Animal and Veterinary Sciences, 1300-477 Lisbon, Portugal; 3 cE3c—Centre for Ecology, Evolution and Environmental Changes & CHANGE—Global Change and Sustainability Institute, Faculty of Sciences, University of Lisbon, 1749-016 Lisbon, Portugal

**Keywords:** antibiotic resistance, virulence factors, captivity, birds of prey, One Health

## Abstract

**Simple Summary:**

Captive birds of prey have played an important role in human history since classical societies, and falconry has been on the List of Intangible Cultural Heritage of Humanity since 2021. In addition to their close relationship with humans, these animals are also in contact with wildlife, as many modern falconry practices depend on this link, such as pest control and hunting. The main objective of this review is to summarize the existing literature on the bacteria found in captive birds of prey and try to understand how these connections affect the dissemination of relevant pathogens in both human and veterinary medicine.

**Abstract:**

Falconry has been practiced for thousands of years and is nowadays frequently employed in activities such as pest control, hunting, falcon racing, and environmental education. Antimicrobial resistance levels have risen in the past years, constituting an emerging global problem with a direct impact on public health. Besides both topics being studied on their own, information on the role of captive birds of prey in the potential dissemination of virulence factors and antimicrobial resistance determinants of bacterial origin is scarce. Multidrug-resistant bacteria, including some extended-spectrum β-lactamase producers, have already been found in several captive birds of prey. Most of the virulence factors found in captive raptors’ bacteria were related to adherence and invasion abilities, toxin production, and flagella. These birds may acquire these bacteria through contaminated raw food and the exchange of animals between keepers and zoological facilities. More studies are required to confirm the role of captive birds of prey in disseminating resistant bacteria and on the routes of interaction between synanthropic species and humans.

## 1. Review Methodology

For this review article, NIH PubMed was used to ensure coverage of the topic. Keywords relevant to this research were identified, enabling the establishment of the search string employed in the initial research process, which included (“Birds of Prey” OR “Raptors”) AND (“Captive” OR “Captivity”) OR “Falconry”, and yielded 239 results. Articles about categories not related to veterinary sciences or microbiology were eliminated. After examining the research titles and excluding articles related to other species or out of the scope of this review, an exhaustive reading of all the abstracts was performed to select the articles to be included. Other references were later obtained to support the information presented, using the snowball search method, obtaining a final number of 101 references.

## 2. History of Falconry

According to the United Nations Educational, Scientific and Cultural Organization (UNESCO), falconry is described as the traditional art and practice of training and flying all species of birds of prey (also known as raptors), now protected as intangible cultural heritage [1]. The role of birds of prey in history and human culture has been well known since classical societies, with representations of birds of prey observed in Sumerian, Greek, and Roman mythology [2]. Besides these religious expressions, falconry as we know it today has been practiced for thousands of years, dating back to 4000 B.C., most likely originating in the Middle East. It largely concentrated on the hunting aspect of the sport, in which hawks, eagles, and falcons would be trained to gather food for human populations [3]. This practice promptly spread throughout Europe and Asia, where these birds began to be raised as a representation of status, being considered that the size and grace of a bird were proportional to its owner’s prestige [4]. Frederick II of Hohenstaufen, the Holy Roman Emperor, wrote the 1240s *De Arte Venandi cum Avibus*, an important treatise recognized as one of the earliest accounts of raptor medicine, in which the role of good hygiene, diet, and exercise in the wellbeing of falconry animals is underlined [5]. The golden age of falconry occurred in the 13th century, right before suffering its first decline with the development and popularization of firearms. By the eighteenth century, only a small nucleus of falconers remained committed to carrying on the tradition of hunting with the aid of these animals [3,6]. 

## 3. Modern Falconry

Nowadays, falconry has found a new revival as novel uses have been uncovered, namely pest management, falcon racing, and environmental education, marking the beginning of a new chapter for this noble activity and expanding the definition of falconry, previously associated with hunting and racing, to the one used today by UNESCO [1,3].

From the very first moment these birds start to be trained for falconry, their behavior changes, and the veterinary approach to them must also differ from that to their wild counterparts [7].

It is important to establish which animals can be defined as birds of prey, as there are no reliable or official criteria for this nomenclature, and the groups included under this umbrella term may vary. In this review, the definition proposed by McClure based on the “ancestral raptorial condition” of the orders was used and includes Accipitriforms (hawks and eagles), Cathartiforms (New World vultures), Cariamiforms (seriemas), Falconiforms (falcons and caracaras), and Strigiforms (owls), with the latter group being composed of nocturnal raptors [8].

The early 2000s saw the beginning of falcon racing, a new sport established in the United Arab Emirates, whose popularity has encouraged the breeding and trading of captive birds of prey worldwide [1,9].

Another relevant use of falconry nowadays is avifauna control in both urban and rural areas, allowing for contact with synanthropic species, most of which are commonly labeled as pests [10,11,12]. These species are becoming more widespread in urban settings as they become adapted to anthropogenic environments and may pose substantial health risks since they have been previously linked to diseases that may be transmitted to humans, along with safety issues [12]. In an attempt to manage this emerging problem, integrated falconry programs have been established with success as an alternative to other pest control strategies, with the benefit of not culminating in animal death as the use of firearms does, but allowing for the establishment of a direct link between these animals and humans [11].

Lastly, falconry can be also employed in educational settings since these animals tend to easily capture attention from the community, establishing an emotional connection and helping organizations to raise awareness and interest in wildlife [13,14].

## 4. One Health: Antimicrobial Resistance

The planet’s sustainability relies on symbiotic interactions between humans, animals, and the ecosystems in which they live, so its challenges must be addressed from multiple angles, promoting closer cooperation and removing academic and professional barriers between these three areas [15]. As a result, the One Health concept was developed, being described as an integrated and unified strategy aimed at achieving a sustainable balance between human, animal, and environmental health [16]. In 2021, Aarestrup’s team emphasized the relevance and necessity of a One Health surveillance program to prevent future pandemics, citing the COVID-19 pandemic as an example of how sensitive society is to these challenges [17].

Antimicrobial resistance constitutes a serious global problem with a major impact on public health, as the use of these compounds is crucial to the safeguarding of both human and animal health. It is considered a critical global threat by the World Health Organization that could kill up to ten million people by 2050 [18], making it vital to tackle this problem by adopting a One Health perspective worldwide. One of the measures that must be applied is the promotion of antibiotic stewardship in the three One Health settings, which has as its main challenges the limited motivation and information of not only health personnel but also the community, the improper use of antibiotics, and insufficient or inadequate establishment of regulatory and monitoring measures in many countries [19].

It is important to understand that antibiotic resistance is a natural phenomenon that occurs even without human interference, as a wide spectrum of antibiotic-resistant genes were identified in environmental bacteria isolated before the discovery of antibiotics [20]. Bacteria can present intrinsic resistance to antibiotics due to their inherent properties, such as the barrier to drug entry found in Gram-negative bacteria, promoted by the outer membrane in their cell envelope [21]. Despite this, the main cause of today’s global crisis is acquired resistance, defined as the resistance gained when previously susceptible bacteria acquire the ability to express a resistance mechanism, which can happen due to mutation or the acquisition of additional genetic material [22].

Some of the proposals for controlling this crisis include increasing the research on promising new strategies to combat these bacteria, such as bacteriophages; the development of synergistic and hybrid antibiotics, enhancing their bioactivity; and education of society about antibiotic use and resistance drivers and consequences. It is indispensable to recall that bacterial resistance and resistance genes are present in a wide range of environments, also including air and migratory bird feces. As such, these ecosystems should be deeply studied to better understand these relationships [23].

## 5. Bacteria Found in Captive Birds of Prey

Despite the centuries-old influence of falconry in our culture, much remains unknown about the bacteria found in captive birds of prey [24]. Although attacks from birds of prey are much less common than attacks from other pets (only 11.6% of cases reported in Qatar were related to falconry, whereas cats were responsible for 53.5% of these cases), these animals can carry a broad spectrum of zoonotic bacteria, making it important to understand the diversity of microbial pathogens that may be transmitted to those in contact with them [25]. Some reports, although quite uncommon when considering the full scope of the observed interactions between these specimens and humans, already show that transmission of zoonotic diseases can occur due to attacks from both wild and captive raptors [25,26], as well as outbreaks of infectious diseases promoted by contact with their pellets [27].

The most common diseases presented by these animal species, as well as the prevalence of agents linked with infectious diseases, are highly influenced by the maintenance of birds of prey in wild or captive conditions and by their use for falconry, education programs, or display in parks and zoological settings [28,29,30,31].

Anthropogenic conditions, coupled with exposure to pollutants found in urban areas, are associated with higher stress levels in raptor species, which trigger a poor body condition and microbiota dysbiosis-related diseases, which can subsequently be worsened by opportunistic pathogens [32]. Captivity is also linked with increased contact with sources of potential pathogens from agricultural, industrial, and urban settings, including multidrug-resistant bacteria, which may be acquired through the ingestion of raw food provided by handlers [27,32,33]. Furthermore, falconers who hunt may feed wild prey to their raptors, which raises the chance of contamination since game birds are important carriers of foodborne pathogens [34,35].

### 5.1. Bacterial Diseases

Despite them being exposed to many diverse bacteria, primary bacterial diseases are not common in raptors in captivity [36].

Pododermatitis is a chronic disease associated with captivity, also called bumblefoot. This condition is associated with poor-quality perches and diet, a lack of hygiene, and exercise and trauma [36,37,38,39,40,41]. The severity of this condition can be intensified by the presence of multiple infectious agents or cardiovascular disorders and, when left untreated, can aggravate and lead to osteomyelitis, septic arthritis, generalized tetanus, and even death [35,36,37,38]. Cardiac diseases in raptors can be frequently caused by bacterial infections, resulting in endocarditis, myocarditis, and pericarditis, and can also be secondary to bumblefoot [30].

*Mycobacterium avium*, the agent of avian tuberculosis, is potentially zoonotic for immunocompromised individuals [36], and one case report has previously described the transmission of the *Mycobacterium avium* subsp. *avium* from infected domestic fowl to raptors raised in a nearby falcon breeding facility [42]. Besides the economic constraints caused by the dissemination of infections by this bacterial species in livestock herds, when transmitted from avian species to humans, *Mycobacterium avium* causes zoonotic and occupational diseases, being often associated with illnesses such as Crohn’s disease, inflammatory bowel diseases, diabetes mellitus, and even immune-related diseases [43].

As for salmonellosis, clinical symptoms are rarely found in captive birds of prey and, when present, are associated with reduced immunity. The transmission of *Salmonella* spp. in captive reproduction facilities has been attributed to shell contamination, ovarian transmission, or the direct infection of chicks with contaminated food [24,36,44,45]. A study conducted in scavenger raptors near urban centers found that birds trapped in rubbish dumps, a more anthropogenic environment, tended toward a higher prevalence of *Salmonella* spp. than birds from the same species living in wild steppes [46]. This study also showed that the most common *Salmonella* serovars isolated from birds in urban areas belong to zoonotic strains and that almost half of the *Salmonella* spp. isolates obtained presented resistance to at least one of the antibiotics tested, with a relevant increase in resistance to quinolones when compared to isolates from animals of the same species living in the wild [47]. Animal carriers of *Salmonella* serovars can transmit non-typhoidal salmonellosis to humans, which is in most cases related to food contamination. However, pets can also act as vehicles of *Salmonella* spp., and although cases of *Salmonella* transmission from pet birds to humans are rare, their potential role as *Salmonella* vehicles increases after contact with wild birds [48].

Despite *Chlamydia psittaci* being previously found in these animals, diseases related to this agent are not often reported [36,49,50]. However, the transmission of this agent to humans can occur through exposition to domestic, pet, and wild birds [51,52,53]. Human psittacosis can either be asymptomatic or lead to respiratory or systemic disease and, if left untreated, can be lethal [54].

Also, *Mycoplasma* spp. can be isolated from tracheal swabs and semen samples, and their relevancy in captivity is connected to the ability of this genus to negatively affect semen quality and diminish artificial insemination success [55,56,57,58].

The antibiotic drugs most used in birds of prey for the treatment of infectious diseases are summarized in Table 1, as well as their respective dose, frequency, and route of administration applied in these birds [36,59]. However, the most important measures for keeping raptor centers free of infectious agents are mainly related to keeping exposure to potential pathogens to a minimum by avoiding contact with wild birds when possible, evading avian-derived food like one-day-old chicks, and applying quarantine and other biosecurity measures to every new animal admitted to a center or to every diseased bird suspected of infectious disease [36].

### 5.2. Microbiome

**Figure 1:**
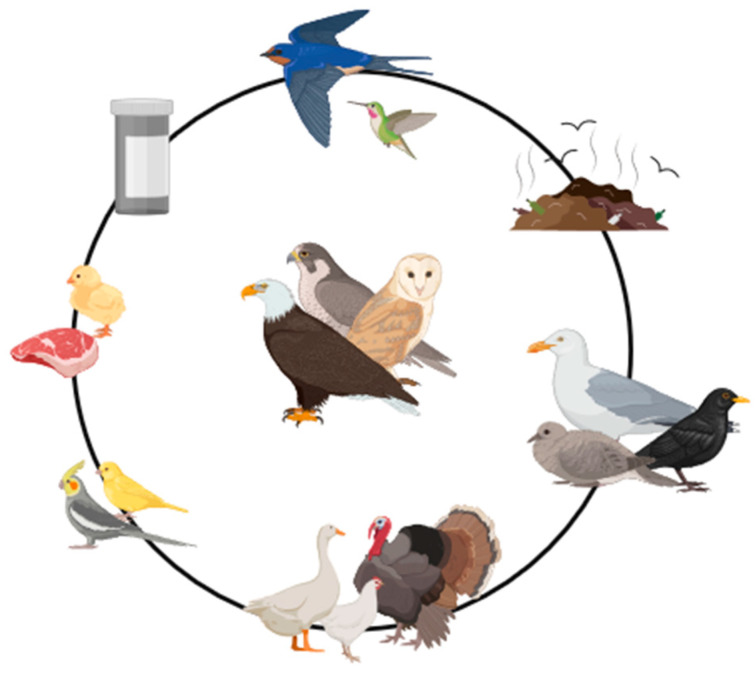
Some variables suspected to alter the microbiome of birds of prey in captivity: contact with wildlife; direct contact with human and animal waste; contact with synanthropic species; contact with domestic waterfowl; contact with other pet birds; diet; and previous exposure to antibiotics. Based on [25,27,32,33,34,35,36,60,61,62,63,64,65] and created using BioRender (https://www.biorender.com/).

Captivity is presently known to affect gut and oral microbiome diversity in birds of prey when compared to their wild counterparts, with observable changes within just one month of direct human contact, and diet is being pointed to as the main factor responsible for alterations in the oral microbiome [60,61,62,63,64,65]. For example, studies have shown that birds who are fed chicken are linked to a wider diversity of Gram-negative bacteria [66], and that the diet commonly provided to captive animals increases the levels of *Salmonella* in falcons [65]. Some other factors responsible for altering the microbiome in birds of prey are also shown in Figure 1.

Resistance rates were proven to be higher in isolates from captive birds of prey than in ones from other zoo birds, and although their prevalence is yet to be determined, the occurrence of ESKAPE pathogens and extended-spectrum β-lactamase (ESBL) producers has already been described in these animals [67]. ESKAPE is an acronym for the group of pathogens presently considered by the World Health Organization (WHO) to be the most relevant as research targets regarding antimicrobial resistance control and includes *Enterococcus faecium*, *Staphylococcus aureus*, *Klebsiella pneumoniae*, *Acinetobacter baumannii*, *Pseudomonas aeruginosa*, and *Enterobacter* spp. [68,69]. As for ESBL production, strains with this ability show resistance to most β-lactam antibiotics and are frequently associated with the failure of antibiotic therapy [70].

A study conducted in a European zoological park described that, in healthy raptors, all the isolates obtained from cloacal and conjunctival swabs were multidrug-resistant [71]. Another study revealed that the most common proteins coded by the genome of isolates from captive raptors were associated with the expression of antimicrobial resistance genes, including β-lactamases and efflux pumps. Most genes associated with antibiotic resistance found in captive vultures were coded for resistance against fluoroquinolones (23.94%), tetracyclines (19.72%), and bacteriolytic beta-lactam antibiotics (19.72%) [72].

Two studies on *Salmonella* spp. showed that bird isolates from this genus presented a high resistance to streptomycin and nalidixic acid [44,45].

Cationic antimicrobial peptides (CAMPs) are known for their microbicidal properties by destabilizing bacterial membranes. Several genes related to CAMP resistance were also found in the gut microbiome of condors [72].

The main hypothesis presented for the high prevalence of multidrug-resistant bacteria in these birds is the contamination of the food given to these individuals, with day-old chicks, rabbits, and mice being the most mentioned food items [27,32,33,45,71]. Other factors may be the frequent shift of birds between enclosures inside the same facility or even their exchange between zoos [60].

To the best of our knowledge, only one study describing the virulence factors present in bacteria from these animals has been published. This work claims that the most significant virulence determinants present within the 1786 associated genes identified were related to bacteria adherence and invasion, toxin production, and flagella [72].

To avoid bacterial transmission from these animals to humans and the environment, bird-keepers should be educated about these potential pathogens and adopt proper hygiene and nutrition practices, as well as protection measures while handling birds of prey, such as the use of falconry gloves, which are of particular relevance since hands are the most frequently injured body parts [25,32]. The implementation of biosecurity measures seems to be the best method of preventing infectious disease outbreaks in captivity facilities [36]. Moreover, more studies are required to confirm the importance of captive birds of prey in resistant bacteria dissemination, as the information available is still scarce.

## 6. Pet Birds as Reservoirs of Resistant and Zoonotic Bacteria

Despite pet ownership being common worldwide, with almost half of European households possessing at least one companion animal, 15.7% of which are birds (the largest group of exotic animals in Europe), awareness of the risks that such close contact with family members pose, especially to immunocompromised people, is not always taken seriously [73,74]. One study demonstrated that even some medical professionals are not aware of the zoonotic risks associated with keeping a pet bird, neglecting important and common zoonotic events, such as the previously mentioned salmonellosis and psittacosis [73].

Even though a lack of knowledge regarding the transmission of infectious diseases from birds to owners is evident in most recent investigations, pet birds have already been indicated as potential reservoirs for zoonotic bacterial agents as dangerous as the aforementioned ESKAPE pathogens, with isolates from pet birds being clustered together with those from their human owners through the determination of bacterial–genetic relationships. As such, it is crucial to monitor pet birds regarding their potential to serve as reservoirs of zoonotic bacterial pathogens to preserve human health [75].

## 7. Impact of Synanthropic Species and Humans: A One Health Approach

Human activity has an essential role in the dissemination of drug-resistant bacteria since domestic, farm, and industrial sewage allows for the accumulation and spreading of antimicrobial resistance genes at a faster pace than other materials [76,77,78]. Antimicrobial-resistant bacteria have reportedly increased in wild animals over the last decade, but the origin of such strains remains unclear, as wildlife is not exposed directly to antibiotics. These strains can emerge due to contact with resistant bacteria or antimicrobial residues present in sewage or domestic animal manure; therefore, the potential of these animal species to act as reservoirs for resistant bacteria should not be underestimated [79,80]. When not in captivity, birds can easily travel long distances between nesting and foraging sites, allowing them to broadly spread antibiotic-resistant bacteria and genes. The fecal resistome of wild birds is highly interconnected with that of their habitat, supporting the idea of their important role in disseminating resistant strains and determinants [81].

Birds of prey are carnivorous, and, when living free, they hunt other animals and avoid human interaction, which makes them important sentinels of the distribution of multidrug-resistant genes in the environment [82]. Several studies have already unveiled the presence of multidrug-resistant bacteria in wild raptors [83,84,85].

On the other hand, synanthropic birds are also known to carry pathogenic and antibiotic-resistant genes, and therefore may spread these genes to other birds and humans due to the close interactions that may occur inside urban ecosystems [86]. Anthropogenic factors have an evident impact on synanthropic species as the exponential growth of the human population’s size is associated with an increase in the frequency of antibiotic-resistant bacteria [87].

In recent years, the occurrence of multidrug-resistant bacteria in synanthropic species, like pigeons [88,89] and gulls [90,91], has been frequently described. A study identified multidrug-resistant *Escherichia coli* strains in synanthropic birds, with the most frequent form of resistance being tetracycline [86]. Another study detected the presence of quinolone-resistant *E. coli* in gulls and established a connection between these strains and the ones isolated from near water habitats, where fecal contamination was detected, suggesting that quinolone-resistant *E. coli* occurring in water may be dispersed by this animal species. The same study also concluded that gulls are important vectors, as most migrate, allowing for antibiotic resistance genes to be spread over long distances [92]. ESBL-producing bacteria have also already been described in these species [93].

As these studies suggest, the transmission of bacteria between wild, synanthropic, and captive species may be possible, although more studies are required to confirm the importance of captive birds of prey in resistant bacteria dissemination and the role they play in interactions between different bird species.

## 8. Disease and Bacterial Transmission Prevention

In captive conditions, hygiene measures are of the utmost importance, with strong evidence corroborating the idea that poor hygiene conditions can lead to a buildup of a wide range of microorganisms in birds’ habitats [94]. Good hygiene procedures are then considered by researchers as a key part of preventing infectious disease [94]. Pathogenic bacteria in aviaries spread over time, with disease transmission being augmented in cases where enclosures are smaller [95].

In closed aviaries, the transmission of bacteria can be controlled by restricting the movements of birds and humans, but this is not possible in the context often found in enclosures with captive birds of prey [94,96]. A preventive health program with accurate records on all transactions and medical conditions should always be established to identify early signs of disease in resident birds, as well as establish quarantine procedures for newly acquired birds, with disease screenings included [96].

When cleaning the spaces dedicated to avian species, it is important to keep in mind that it is extremely challenging to truly disinfect enclosures with vegetation, with exposure to both rain and sunlight being a good measure to reduce the number of pathogens in these types of aviaries [94]. At least once a year, the enclosures should be examined for the need for more in-depth cleaning, and materials that can easily become contaminated, such as logs and bark, should be replaced [94].

All leftover food should be removed at least once daily, ideally at the same time as water changes are also performed [94]. As previously mentioned, food can be an important vector of pathogenic bacteria and should always be checked for contamination and spoilage before storage, and good hygiene practices should be applied when meal prepping to avoid cross-contamination [64,65,96].

Proper hygiene can protect both animals and handlers and can be sufficient to stop outbreaks of, for instance, salmonellosis [94]. It is important to encourage prophylactic measures for those directly handling these birds and their products, starting with immunization against tetanus [94]. When working with birds, using protective gloves and washing hands reduces the risk of handlers contracting zoonotic diseases [97]. If possible, keepers could also benefit from the use of specific shoes and clothing when entering these enclosures or when handling these birds [98]. All professionals in contact with birds should be informed of the potential health risks and be educated on how to avoid and detect early signs of disease [99].

Nowadays, due to their common use, it is difficult to reduce contact between these falconry birds and both wild birds and humans; therefore, these animals should be subjected to screening tests more frequently than other birds [99]. Important pathogens, such as *Yersinia pseudotuberculosis*, can also be introduced into aviaries by rodents, either acting as mechanical or biological vectors, which should and can be avoided when planning the construction of these spaces [98,100].

Together, good nutrition, management, and husbandry practices should be sufficient to maintain the health of captive species [96]. When needed, the prescription of any antimicrobial compounds should be exclusively carried out by a veterinarian. These health professionals must opt for antibiotherapy after performing a careful evaluation of each clinical case, which must include an accurate diagnosis and, if possible, susceptibility tests for the associated bacteria [98,101].

## 9. Conclusions

Captivity for birds of prey affects microbiota diversity and has been related to high rates of antibiotic resistance, with the main reason presented being the transmission of bacteria through raw food. Despite some reports on human infections being transmitted by these birds, much remains unknown about how the contrary can also happen and how contact with humans or even synanthropic species can promote this dissemination.

Prevention seems to be the key factor in controlling this eminent issue, with biosecurity measures being essential when it comes to stopping the spread of zoonotic pathogens. Awareness and education on the importance of antimicrobial resistance and how to reduce its impact on our ecosystems are also necessary for those directly in contact with these birds.

As such, the main conclusion from this review is that additional research is needed to understand the importance of captive birds of prey in resistant bacteria transmission and how they interact with synanthropic animals and humans, as the information on this link is still very limited.

Possible future directions to improve our understanding regarding falconry birds include (1) the design of studies focusing on samples from both falconry birds and their handlers being evaluated in parallel; (2) the development of more studies on the virulence traits present in the microbiome of captive birds of prey; and (3) the design of comparative analyses between the resistance genes detected in the food supplied to birds of prey and those identified within the gastrointestinal microbiota of these same animals.

## Figures and Tables

**Table 1:** Antibiotic drugs commonly used in captive birds of prey [36,59].

Antibiotic	Dosage and Administration	Reference
Amikacin	15–20 mg/kg i.m. q24 h	[59]
Amoxicillin	150 mg/kg i.m. q24 h 150 mg/kg orally q12 h	[36]
Amoxicillin/Clavulanate	150 mg/kg orally 150 mg/kg i.v. q12h or i.m. q24 h	[36]
Azithromycin	50 mg/kg orally q24 h 5 days for Chlamydophilosis	[36]
Cefalexin	40–100 mg/kg i.m. or orally q6–8 h	[59]
Cefazolin	50–100 mg/kg i.m. or orally q12 h	[59]
Clindamycin	100 mg/kg orally q24 h	[36]
Doxycycline	50–75 mg/kg orally q12 h 100 mg/kg i.m. for 5–7 days	[36]
Enrofloxacin	15 mg/kg orally or i.m. q12 h	[36]
Marbofloxacin	10–15 mg/kg i.m. or orally q12–24 h	[59]
Gentamicin	2.5 mg/kg i.m. q8 h	[59]
Oxytetracycline	16 mg/kg i.m. q24 h in great horned owls	[59]
	48 mg/kg i.m. q48 h in owls 25–50 mg/kg i.m. or orally q8 h for 5–7 days 50–200 mg/kg i.m. q3–5 days	

Legend: intramuscular (i.m.); intravascular (i.v.); each (q); hour (h).

## Data Availability

The datasets used and analyzed during the current study are available from the corresponding author upon reasonable request.
